# Parents’ experiences of sham feeding their child with esophageal atresia at home while awaiting reconstructive surgery. A qualitative interview study

**DOI:** 10.1007/s00383-024-05660-w

**Published:** 2024-02-29

**Authors:** AnnaMaria Tollne, Tuva Nilsson, Jan F. Svensson, Markus Almström, Elin Öst

**Affiliations:** 1 Pediatric Surgery Unit, Department of Pediatric Surgery, Karolinska University Hospital, Astrid Lindgren Children’s Hospital, 171 76 Stockholm, Sweden; 2 Department of Women’s and Children’s Health, Karolinska Institutet, Stockholm, Sweden

**Keywords:** Esophageal atresia, Delayed reconstructive surgery, Sham feed, Homecare, Parents’ experience

## Abstract

**Purpose:**

This study aimed to explore parents’ experience of sham feeding their baby born with esophageal atresia at home, waiting for reconstructive surgery.

**Method:**

Semi-structured interviews were conducted with parents of six children born with esophageal atresia waiting for delayed reconstruction. The interviews were analyzed using qualitative content analysis.

**Results:**

Parents experienced that sham feed reinforced the healthy abilities in their baby. They had faith in their own ability as parents to care for their child as well as to see to their baby’s strength to cope with difficulties. Parents expressed that the health care system can hinder as well as be a major support on their way to a more normal life at home while waiting for reconstructive surgery.

**Conclusion:**

The experience of sham feeding at home while waiting for reconstructive surgery is characterized by positive aspects both for children born with esophageal atresia and their parents.

**Supplementary Information:**

The online version contains supplementary material available at 10.1007/s00383-024-05660-w.

## Background

Esophageal atresia (EA) is a rare congenital malformation where the child is born without a continuous esophagus, and hence with no connection from the mouth to the stomach. The incidence is 2.43/10,000 children and EA is often associated with other congenital malformations such as cardiac defects, other gastrointestinal anomalies, as well as limb and urinary tract malformations [1]. Survival rates has increased over the last decades to 90–95% today [2, 3]. A child with EA needs surgery in the first days of life to gain access to the gastrointestinal tract for feeding orally. In a majority of cases, a primary anastomosis is performed whereby the two ends of the esophagus are connected.

About 10% of children born with EA will have a delayed reconstruction surgery due to long-gap esophageal atresia (LGEA), with prematurity or malformations increasing the risks with surgery for this problem in the babies’ first days of life [4]. These children can have extensive care needs and need to stay in the hospital for months, sometimes up to a year, waiting for surgery with risk of problems with oral eating, specifically with swallowing and chewing after the reconstructive surgery [5–8].

It is known that early oral feeding promotes swallowing abilities and also accomplishes pharyngo-esophageal coordination, and further protects airways during deglutition [9, 10]. To enable early training and eating, sham feeding can be an option. Sham feeding is a procedure whereby the child is fed orally even though the child does not have a continuous esophagus. When an infant is being sham fed, he or she can either be bottle or breast fed. When the baby swallows the milk or formula, a nurse or trained parent aspirates the milk via a syringe connected to a tube in the upper pouch of the esophagus. The same milk is then given in the gastrostomy with a syringe [11]. It is important that the procedure is done when the baby is fully awake, shows signs of hunger, and is willing to eat in order to reward the feeling of being hungry with eating and being full.

Feeding problems in children with EA is not only a nutritional issue, since it has been shown that parents have concerns related to feeding/swallowing difficulties. Mothers of infants born with EA and having feeding problems have previously reported higher levels of anxiety than mothers of healthy infants [12], and mother–infant interaction might be affected during feeding because of feeding difficulties [13]. At the same time, parents’ ability to interact with the child during feeding has been described to have a positive influence on the quality of the mother–child interaction [14]. Another previous study indicates that the time span before oral feeding starts is associated with concern, and that a correlation exists between late start and increased concern [15]. Also, parents of children with EA have reported reduced mental health compared with the general population, suggesting that parents may experience substantial emotional burden [12, 16]. A pilot study showed that mothers who had been sham feeding their child after bowel surgery had reduced feelings of stress [11]. Previous research by Aziz et al. has shown that in selected cases, it can be safe to care for these children at home before reconstructive surgery [17].

In Sweden there are two pediatric surgery centers treating children born with EA, whereof Karolinska University Hospital in Stockholm is one. In the course of the last 5 years, 15–23 infants yearly with EA have undergone surgery at Karolinska University Hospital, whereof 1–5 infants a year had delayed surgery due to LGEA, prematurity, or other congenital malformations that would have increased the risks with surgery in the babies’ first days of life. These infants almost always have a delayed primary anastomosis (DPA). In rare cases some infants have an unusually long gap and are offered a jejunal interposition or gastric pull-up when a DPA is not an option. These are performed with open surgery, according to protocol in Stockholm, when the infants are between the ages of 1 and 13 months. In 2018, parents to infants with EA were for the first time offered the option of going home and caring for their baby themselves while waiting for reconstructive surgery in Stockholm. In 2020 a new program was implemented at Karolinska, wherein parents of children eligible for sham feeding at home were offered structured training before discharge to care for their child and do the feeding by themselves. They were taught CPR for infants, feeding and gastrostomy care, oral suctioning of saliva, inhalation with a nebulizer, respiratory physiotherapy, and monitoring of saturation. They also learned to change the tube in the upper esophageal pouch in case it was pulled out by accident and to manage the mobile, digital continuous suction connected to the tube in the upper pouch. All stages of progression were documented in the child’s medical record.

The aim of this study was to explore parents’ experience of sham feeding at home of their baby born with esophageal atresia, while waiting for reconstructive surgery.

## Method

### Design

In this qualitative study, inductive content analysis was performed to explore parents’ experiences [18].

### Setting and recruitment

To begin with, all patients born with EA treated at Karolinska University Hospital Stockholm, and who had been sham feeding at home before reconstructive esophageal surgery between 2018 and January 2023, were identified in the electronic patient record (EPR). All families (*n* = 8) were contacted by phone by one of the authors (TN) and were given information about this study. The author who contacted the families had not been in the nursing team prior to this study. After getting informed consent, parents were offered an interview by speaker phone or digitally via link. The parents could choose if they wanted to do the interview together or if only one of the parents should participate.

### Data collection

All interviews were conducted over speaker phone in the parent’s native language (Swedish in all cases) by one author (TN). The first interview was conducted as a pilot to evaluate the interview guide, and since the guide was not revised, the initial interview was later included in this study. Every interview adhered to a semi-structured guide with background questions and open-ended questions about parents’ experience of sham feeding and caring for their child at home before reconstructive surgery. The interview guide was developed for this study (Appendix 1). The interviews lasted 20–55 min, were digitally audio recorded, and were transcribed verbatim into text files by three of the authors (TN, AMT, and EÖ).

### Analysis of data

The data were analyzed with content analysis, following the approach taken by Elo and Kyngäs [18]. To achieve dependability the results were analyzed by three of the authors (AMT, TN, and EÖ). After reading through the text files of the interviews several times, the text was coded and the material was sorted into groups. Then the groups were abstracted into subcategories, and subcategories with similar content were abstracted further into generic categories that formed one main category combined. The three authors discussed every step of the analysis until agreement was reached.

### Ethical considerations

Ethical approval was granted by the Swedish Ethical Review Authority, register numbers 2023–00384-01 and 2023–02649-02. The parents were contacted over the phone and given information about this study verbally and in writing (sent by post), and were able to ask questions about this study. Written informed consent was obtained.

## Results

Six families (*n* = 6) agreed to participate in this study. Four interviews were held with mothers only and two with both parents (*n* = 8). By the time of the interviews, the children were from 1 month to 4 years of age and had a history of awaiting reconstructive surgery from one to seven months. For demographic data, refer to Tables 1 and 2.

**Table 1:** Demographic data of parents

	*n*	Age, median (range)	Experience of older sibling	Experience of breastfeeding	Desire to breastfeed after delivery	Parents who lived together in time of birth	Parents who lived together in time of the interview
**Parents**	8	36 (29–42)	4	2	5	7	7
Fathers	2	34 (33–35)	1	0	0	2	2
Mothers	6	36 (29–42)	3	2	5	5	5

**Table 2:** Demographic data of children

	*n*	Age in months, median (range)	Months waiting for reconstructive surgery Median (range)
**Children**	6	30 (1–48)	4 (3–7)
Girls	3	48 (1–48)	3.5 (3–4)^a^
Boys	3	24 (24–36)	4.3 (3–7)

^a^One child (*n* = 1) waiting for reconstructive surgery

The qualitative content analysis resulted in one main category, four generic categories, and twelve subcategories. All the categories are presented in Table 3 and with quotes from the parents in the text below to illustrate the result.

**Table 3:** Subcategories, generic categories, and main category

Subcategories	Generic categories	Main category
To bond with their child	To see the healthy abilities	Sham feeding at home reinforce the healthy abilities in the babies
Manage to sham feed in everyday life		
Sense of safety of one’s own home		
The desire to live a normal life		
Sham feed influence on the ability to eat	Resources and skills	
Trust in their Childs’ driving force		
Trust in the parenting ability		
Information before sham feed	Health care that facilitates	
Health care as support		
Complications dim the experience	Hindrance	
Concern before discharge		
Health care as hampering		

## Parents experienced that sham feeding reinforced the infants’ healthy abilities

All parents were grateful for having sham fed their children and expressed a belief that sham feeding had an impact on how well the child was able to eat after reconstructive surgery. The parents talked about similarities with healthy siblings and described a feeling of trust in their child’s own abilities. Some situations at home had been described as resource demanding and challenging, but the parents also described how they managed to find techniques to create opportunities for sham feeding at home. Most of the parents described a feeling of confidence in their own or in their partners’ parenting abilities before it was time to leave the hospital. But even the parents who had doubted their abilities and expressed that they were worried before discharge proved themselves wrong after they had settled at home. The parents described health care personnel as generally supportive in taking care of their baby but also that the personnel could at times be a hindrance when they wanted to sham feed and take care of their baby. All the parents were asked if they have experienced any adverse events at home and if they knew what to do if anything would happen. Only one of the parents had experienced an adverse event when they had sham fed their baby at home, but none that required calling in medical support or going to the hospital.

### To see healthy abilities

All parents expressed that sham feeding their baby contributed to the feeling of closeness and normality. They talked about a *sense of safety in their own home* and a strong *desire to live a normal life. Parents’ sense of connection with their child* and how they *managed to sham feed in everyday life* contributed to bonding and confidence in the mother–child team.

It [sham feed] is a little more intimate…For me, it does a lot for the connection. Absolutely. You get the feeling that it´s actually a completely normal baby, just that she´s a little sick.—Interview 6.

Some parents felt that it was sometimes difficult to sham-breastfeed, but that it was easier for the child to take the bottle instead. Some mothers stated that they managed to sham feed by themselves, without the help of a partner, which in turn had made everyday life easier. The parents had the ability to find solutions and techniques to cope with sham feeding on their own because there was a strong desire to sham feed at all meals, even at night when the baby was awake and was hungry. It turned out that the parents thought it was nice to do this in their own way.

Then, of course, sham feeding requires its resources, to put it that way. So, it was a little more difficult…Like in the beginning we thought that we both needed to be present to do it [sham feeding], but then I found a technique where I put my son in a baby bouncer and then I fed him with one hand and pulled the syringe with other hand and…well…tricks like that. So, we found a good way…And we were very thorough with sham feeding him every meal when he was awake.—Interview 2.

Other parents stated that sham feeding was a two-person job and that it could be stressful to integrate sham feeding into everyday life, but that there was satisfaction when they could squeeze it in. Being at home also created an opportunity for siblings to be involved and for sham feeding to be a part of everyday life. The positive feeling about coming home was something that all interviewed parents shared. However, the feelings before going home could vary somewhat, with some parents expressing that they needed encouragement from health care personnel to go home and begin to trust their own abilities.

At the same time, I know that it`s good to come home. I know we needed that. I was terrified, but at the same time I needed a push to go home because I needed to start being able to trust myself.—Interview 3.

Parents stated that even before going home, they had a feeling that they were ready, and that the desire to go home motivated them to learn to sham feed and to learn other nursing skills. A normalized situation had been created for the parents to care for their child with extensive care needs. They described that it felt safe to sham feed at home knowing they could contact the pediatric surgery ward if necessary.

Furthermore, there was a wish to live as usual when the family returned home, to have a normal family life, even if this was not always possible. There was a desire to normalize things for the child being sham fed. One parent expressed the opinion that sham feeding felt like a normal way to feed their child. It was a positive experience. The parents treated their child as if it was healthy and saw that the sham-fed child had the same kinds of abilities as healthy siblings also had had as infants. Although expectations did not always match reality, sham breastfeeding provided a sense of normalcy.

You thought that when you came home, you would come home with a healthy child, but that was not the case. You had to bring all the machines, medicines…Yes, you packed an entire hospital with you when you went somewhere. But at the same time, I probably also decided that once we got home that I didn’t want to limit myself just because we had a sick child, but I chose to accept that it is what it is and that we can take everything with us and continue living as usual.—Interview 1.

Some parents said they had the belief that their children would have been perceived as sicker if they had to be hospitalized while waiting for reconstructive surgery.

### Resources and skills

In all of the interviews, parents were united in the belief that *sham feeding influenced the ability to eat* after reconstructive surgery and also children’s present ability to eat. The parents had *trust in the child’s driving forces* and also *trust in their own parenting skills*.

I am one hundred percent sure that it has made my son eat completely unhindered today.—Interview 2

One parent expressed that their child had been taught, and learned to, eat from the start and that the child’s learning curve was similar to that of a healthy child. Sham feeding had normalized the child’s eating behavior. Parents described that the child was eager to start eating after reconstructive surgery and that the child knew how to eat and started to eat freely after surgery.

A belief in the child’s own abilities was something that was expressed in several interviews. One parent said that waiting for surgery was a positive experience since the child, in the meantime, had grown and become stronger. Another parent shared how well their child had coped with sham feeding and immediately understood how to breastfeed or take the bottle. Some parents related that how their child had clearly shown a willingness to eat, that the child had managed it, and that there were similarities with healthy siblings, such as the ability to cough and protect the airway if the child accidently swallowed wrong.

Several parents had a feeling of trust and confidence in their own parenting skills. Being active as a parent in the care of the child in the hospital contributed to an increased feeling of security and confidence in their own parenting ability. Two parents said that they themselves had felt insecure about going home but that their partner had felt confident and ready. They trusted their partners’ ability to care for their child.

But the fact that I felt safe in the situation made you feel safe. Yes…So I think we felt that we had the situation under control. Or – we had it.—Interview 5

There was a feeling of confidence and optimism that made the parents ready to take their child home.

### Hindrances

There were situations where *complications dimmed the experience* of sham feeding. For example, these could include complications due to other congenital malformations or problems, such as a leaking gastrostomy or a stoma difficult to bandage. Different nursing needs required focus and energy from the parents who needed to priorities and could therefore not always focus on sham feeding. Complications leading to hospital admission contributed to parents’ concern.

… There were very few times [we could sham feed] because, I think there was so much at home so it wasn’t even that I… we focused on.—Interview4

Some of the parents expressed *concern before discharge*. The concern was related to sham feeding by themselves at home, about whether they could use the technical equipment (despite proper training), and to being at home while also having to be prepared that something could happen. They were worried that something would go wrong and that the child would aspirate breast milk or formula into the lungs.

They said we believe in [you]… And my partner said that ‘I trust myself—If he chokes, I know what to do’, but I’m not like that. I just get really scared and see the worst-case scenario instead. We are very different in that way.—Interview 3.

Several parents talked about the *health care system as a hindrance* when the nursing staff sometimes lacked competence regarding sham feeding and that parents could not always get help in sham feeding when their baby was awake and was hungry. In some cases, the parents felt that they had great confidence in their abilities to care for their child, but that health care professionals rather held them back, which resulted in the parents’ feeling controlled and distrusted. In these cases, it was perceived that sham feeding took place on the basis of health care personnel’s premises.

I have felt calm…that I feel safe in taking care of my child…It was more that it sometimes felt like they [nursing staff] didn't trust me to fix it all by myself. I felt…what can I say…It’s so hard to explain but I think I felt a little distrusted…maybe. Because I felt that I had the situation under control.—Interview 1.

A couple of parents also expressed that they experienced sham feeding as a private moment with their baby and that it was difficult to keep it that way when a lot of nursing staff would be present. It could be a stressful feeling to be observed, but at the same time there was a sense of security in knowing that help was available. Some parents had experienced the feeling of not being able to live up to health care personnel’s expectations regarding sham feeding and had the desire for more support to be able to care for their child themselves.

### Health care that facilitates

Several parents stated that they received *information about sham feeding* prenatally, when they visited the pediatric surgery ward. The information came from both surgeons and nurses. On the other hand, information was difficult to remember after the child was born. It was hard to imagine before the baby was born how things actually were going to be. Depending on the mother’s or the baby’s health after delivery, information postpartum was also difficult to remember.

But they explained somehow that he will…I won’t be able to breastfeed or I will be able to breastfeed later […] but I don’t think I took it in at all…It kind of came later. So I probably got all the information at the ward. I just don’t remember so much of it.—Interview 1.

Some of the mothers expressed concern and sadness after being told about the diagnosis prenatally, as well as a specific grief about not being able to breastfeed their child.

When we found out it was esophageal atresia, the first thing we were told [at the general pediatric hospital they first visited] was that I wouldn’t be able… or that what we heard anyway…to breastfeed her and it felt unbearable! That was almost the hardest thing there in the beginning.—Interview 6.

In all the interviews, parents talked about the importance of *the health care system as a source of support.* Parents described a secure and confident overall feeling about the health care system. Some parents felt safe at the hospital where help was near all the time. The parents felt that they needed encouragement from health care professionals to trust their parenting skills and encouragement to dare to go home. One parent described a very good relationship with the nursing staff, and that the child's progress was celebrated together with them. They also felt a sense of safety knowing where to call when they were discharged. They felt supported when they were sham feeding their child.

I became friends with them [nursing staff] at the hospital, I lived there for so long. So as soon as it became something…ah…”but now she could eat” or “now… now she pooped” or whatever it was, we had a party. We celebrated her (laughs). Well…they were always there, I… It was… I got a lot of support.—Interview 4.

Two families emphasized that access to a lot of information and education provided a sense of support, and that this was important to create a sense of security before returning home.

## Discussion

The present study has explored the experiences of parents who sham fed their baby born with EA at home while waiting for reconstructive surgery. The analysis results in the main category *sham feeding at home reinforces healthy abilities in the babies.* All parents were grateful that they had sham fed their children and believed that sham feeding impacted how well the child was able to eat after reconstructive surgery. The results point to the importance of parental participation, information for the parents, and the importance of the role which health care personnel plays in the process of teaching the parents to sham feed. Results also indicate the importance of staff seeing to other necessities to make it possible for the parents to continue caring for their children at home while awaiting reconstructive surgery.

Previous research has indicated that children born with EA, who need to wait for reconstructive surgery, can be cared for at home with assistance from home care [16]. The results of this study suggest that this is possible without home care assistance, and in addition, the results of this study indicate that the parents experience it as safe and strengthening for the child and family to be at home while waiting for reconstructive surgery and that sham feeding at home also is something that contributes to this experience.

The parents talked about a sense of safety in one’s own home and a strong desire to live a normal life. All parents expressed that sham feeding their baby contributed to the feeling of closeness and normality. Previous research has shown that families often prefer care at home to hospital-based care [19–21]. Care at home can strengthen and normalize everyday family life [19, 22–25]. It has also been shown that families with a severely ill child strive to gain control over their lives by keeping the family together and maintaining normal family life [26]. However, long or repeated hospital stays are a threat to this [27]. In this study parents expressed a sense of connection with their child, and related that how they managed to sham feed in everyday life contributed to bonding and confidence in the mother–child team. Being at home also allowed siblings to be involved and sham feeding to be a part of everyday life. Being active as a parent in the care of the child in the hospital contributed to an increased feeling of security and confidence in one’s own parenting ability. Facilitating the opportunity for every parent to understand their child’s situation and be the primary caregiver can build trust and readiness for transferring full responsibility to the parents [28–30]. Both formal and informal education have been presented as successful when preparing parents for discharge, but it is important to consider each parent’s unique needs [31, 32]. Children should only be admitted to hospital when care cannot be provided in any other place, if the care they require cannot be equally well provided at home or on an outpatient basis [33].

Although the preferred strategy of our protocol in Stockholm is to avoid dissection of the upper esophageal segment until the delayed primary anastomosis and use an esophageal tube to drain saliva from the upper pouch, we believe that similar approach to sham feed could be used in the case of an esophagostomy where the milk would be collected in the stoma bag and re-fed into the gastrostomy. This has been previously described by Kimura and co-workers [34].

This study does not provide any long-term data on feeding habits or difficulties for afflicted children later in life, but other studies have shown that children born with EA, LGEA in particular, are at risk for becoming dependent on partial or full enteral tube feeding, which in turn can cause severe strictures [35]. It has also been shown that digestive problems have a negative impact on health-related quality of life [36]. In our study parents expressed a belief that sham feeding had normalized the child’s eating behavior and that the child was eager to start eating after reconstructive surgery. This is only the results of a few family’s experiences, but it indicates that to sham feed your child at home before reconstructive surgery could help normalize eating behavior in children with EA. Further studies are needed to investigate long-term effects of early sham feeding.

### Limitations

There are several limitations that need to be considered in this study. This study was conducted on a small group consisting of parents of six children. The result reflects their experiences and the transferability should be considered carefully. To our knowledge there are no other similar studies published that can either confirm or reject our result. One other aspect that needs to be highlighted is the criticism that emerged during the interviews, mainly regarding the health care system as a hindrance. These results are important and should be seen as areas of improvement for our unit.

## Conclusion

The experience of sham feeding at home while waiting for reconstructive surgery has positive aspects, both for children born with esophageal atresia and their parents.

## Supplementary Information

Below is the link to the electronic supplementary material.

Supplementary file1 (DOCX 20 KB)

## Data Availability

No datasets were generated or analyzed during the current study.
