# Supplementary material for: Parents’ experiences of sham feeding their child with esophageal atresia at home while awaiting reconstructive surgery. A qualitative interview study
Source: Pediatr Surg Int. 2024 Feb 29;40(1):61. doi: 10.1007/s00383-024-05660-w (PMC10904505; doi:10.1007/s00383-024-05660-w)
Supplement: Supplementary file 1 — Supplementary file1 (DOCX 20 KB) [file 383_2024_5660_MOESM1_ESM.docx]

| *Appendix 1: Interview guide* |
| --- |
| Background information |
| - Your child is born with Esophageal atresia. Did you need to wait for the surgery? Do you remember how long? |
| - Did you know before your child was born that he/she had Esophageal atresia? |
| - How old is your child today? |
| - How old are you? |
| - What was your family situation like when your child was born? How does it look like now? |
| Thoughts about feeding your child and siblings |
| - During your pregnancy, did you think about how you were going to feed your child after it was born? |
| - How was your experience of feeding siblings? |
| Sham-feed |
| - How did you receive information about sham-feed? |
| - How was it when you started to sham-feed? |
| - How was it to go home and sham-feed? |
| - Did you feel ready to go home? |
| - Was there anything that worried you? |
| - Was there any adverse event at home? |
| - If so, did you know what to do? |
| - Was there any other problem at home before surgery? |
| - Do you think that you sham-feeding your child at home before surgery had any effect on you and your child? |
| - If yes, how so? |
| - How did you experience your support from the hospital? |
| - Is there anything else you want to add? |
